# Synovial Fluid Characteristics and Pain Recovery Trajectory Following Rehabilitation in Patients with Meniscal Tears: A Retrospective Cohort Study

**DOI:** 10.3390/healthcare14070962

**Published:** 2026-04-06

**Authors:** Murat Baloğlu

**Affiliations:** Division of Physical Therapy and Rehabilitation, Gazi Yaşargil Training and Research Hospital, Health Science University, Diyarbakır 21280, Turkey; murbal21@hotmail.com

**Keywords:** meniscal tear, rehabilitation, synovial fluid, erythrocyte positivity, WORMS, inflammatory phenotype, knee pain

## Abstract

**Background:** Meniscal tears are a common cause of knee pain and functional limitation, yet determinants of pain recovery following rehabilitation remain incompletely understood. Structural imaging findings often show limited correlation with clinical symptoms. This study aimed to evaluate longitudinal pain trajectories after rehabilitation in patients with meniscal tears and to investigate whether synovial fluid characteristics and structural joint degeneration are associated with pain improvement. **Methods:** This retrospective cohort study included 59 patients with meniscal tears who underwent structured rehabilitation. Structural degeneration was assessed using the Whole-Organ Magnetic Resonance Imaging Score (WORMS). Synovial fluid cytology evaluated neutrophil predominance (PNL) and erythrocyte positivity. Pain intensity was measured using the Visual Analog Scale (VAS) at 3 months, 6 months, and 1 year. Longitudinal changes were analyzed using the Friedman test, and predictors of pain improvement (ΔVAS from 3 months to 1 year) were evaluated using multivariable linear regression. **Results:** VAS scores decreased significantly over time (*p* < 0.001), indicating sustained pain reduction during follow-up. In the multivariable regression model (F(4, 54) = 2.80, *p* = 0.035), 17% of the variance in pain improvement was explained (R^2^ = 0.17). Synovial erythrocyte positivity was modestly associated with greater longitudinal pain reduction (β = 0.75, 95% CI 0.15–1.36, *p* = 0.016). Age was also a significant predictor (β = 0.025, *p* = 0.043), whereas WORMS score and PNL positivity were not significantly associated with pain improvement. **Conclusions:** Pain recovery following rehabilitation in patients with meniscal tears appears to be influenced more by intra-articular biological characteristics than by structural imaging severity alone. Synovial erythrocyte positivity may indicate a potentially reversible inflammatory phenotype associated with higher early pain but greater subsequent improvement. These findings support a multidimensional model of knee pain and suggest that synovial characteristics may help improve clinical risk stratification during rehabilitation planning.

## 1. Introduction

Meniscal tears are among the most common knee injuries encountered in clinical practice and represent a significant source of pain, functional limitation, and healthcare utilization [1]. Epidemiological studies indicate that meniscal injuries occur across a broad age spectrum, ranging from acute traumatic tears in younger individuals to degenerative tears in middle-aged and older populations [2,3]. Regardless of etiology, persistent knee pain and impaired functional capacity remain the primary reasons for seeking medical care. Although surgical and non-surgical management strategies continue to evolve, structured rehabilitation remains a cornerstone of treatment, particularly for patients managed conservatively or following arthroscopic intervention [4,5]. Pain reduction and functional recovery are primary therapeutic goals, yet considerable interindividual variability exists in rehabilitation outcomes [6].

Beyond structural joint damage, emerging evidence suggests that the intra-articular microenvironment may influence symptom severity and recovery trajectories [7]. Synovial fluid composition reflects ongoing inflammatory and hemorrhagic processes within the joint and may provide insight into the biological phenotype of meniscal injury [8]. Acute meniscal injury is frequently accompanied by synovial activation, cytokine release, and infiltration of inflammatory cells, which can amplify nociceptive signaling and contribute to pain perception [9,10]. Neutrophil predominance and the presence of erythrocytes in synovial fluid have been associated with acute inflammatory responses, tissue irritation, and nociceptive activation [11]. These findings may reflect a transient inflammatory or hemorrhagic intra-articular environment following injury. However, the extent to which such synovial fluid characteristics influence mid-term rehabilitation outcomes remains insufficiently characterized [12].

Structural joint degeneration, commonly evaluated using imaging-based scoring systems such as the Whole-Organ Magnetic Resonance Imaging Score (WORMS), is another important determinant of knee pain [13,14]. Nevertheless, structural abnormalities alone do not consistently correlate with symptom severity or recovery potential [15,16]. Several studies have demonstrated a discordance between imaging findings and patient-reported symptoms in knee pathology, suggesting that structural degeneration alone cannot fully explain the variability in clinical presentation [17]. This discrepancy underscores the need to explore additional biological markers that may better explain variations in pain trajectories during rehabilitation [18].

Early identification of patients who are likely to experience rapid improvement versus those with delayed recovery may facilitate personalized treatment planning, resource allocation, and expectation management [19]. In particular, the identification of biological markers reflecting the intra-articular environment may help clinicians better understand the mechanisms underlying pain persistence and recovery.

Therefore, the aim of this retrospective cohort study was to evaluate longitudinal pain trajectories over a one-year follow-up period in patients with meniscal tears undergoing rehabilitation and to investigate whether synovial fluid findings and structural degeneration (WORMS score) are associated with mid-term pain improvement.

## 2. Materials and Methods

### 2.1. Study Design and Population

This retrospective cohort study included patients diagnosed with meniscal tears who underwent structured rehabilitation and had available synovial fluid analysis and follow-up pain assessments. The study was conducted in accordance with the Declaration of Helsinki and approved by the Clinical Research Ethics Committee of Gazi Yaşargil Training and Research Hospital (Approval No: 698, Date: 5 March 2021). Patient data were obtained from electronic medical records and institutional clinical databases. Demographic information, clinical characteristics, MRI findings, and synovial fluid cytology results were retrospectively extracted and verified for completeness. All clinical assessments and procedures were performed as part of routine clinical care.

During the study period, 92 patients with meniscal tears were initially assessed for eligibility. Patients were included in the study if they met the following criteria:

(1) Meniscal tear confirmed by magnetic resonance imaging (MRI);
(2) Participation in a structured rehabilitation program;
(3) Availability of synovial fluid cytological evaluation obtained during diagnostic or therapeutic arthrocentesis;
(4) Availability of complete pain follow-up data measured using the Visual Analog Scale (VAS) at 3 months, 6 months, and 1 year after initiation of rehabilitation.

**Figure 1:**
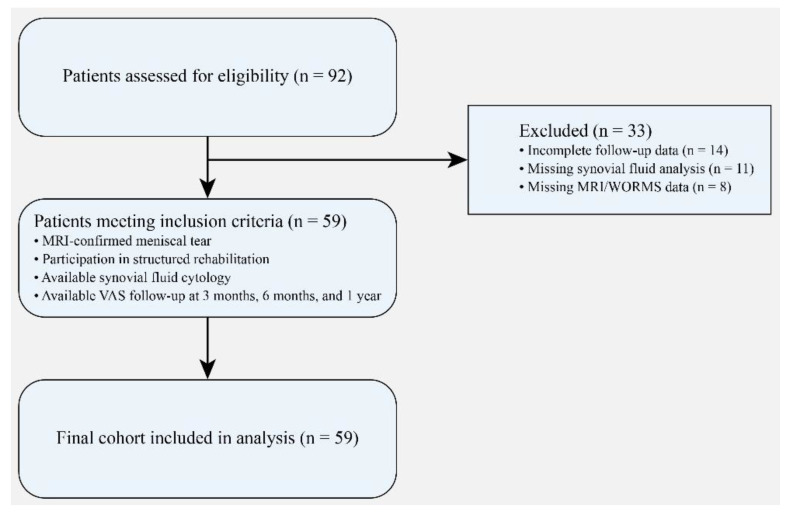
Flow diagram illustrating the patient selection process in accordance with STROBE recommendations for observational studies. A total of 92 patients with meniscal tears were initially assessed for eligibility. Thirty-three patients were excluded due to incomplete follow-up data (*n* = 14), missing synovial fluid cytology (*n* = 11), or missing MRI/WORMS data (*n* = 8). After applying the predefined inclusion and exclusion criteria, 59 patients were included in the final cohort and analyzed in the study.

Patients were excluded if they had incomplete follow-up data (*n* = 14), missing synovial fluid analysis (*n* = 11), or missing MRI/WORMS data (*n* = 8). After applying the inclusion and exclusion criteria, 59 patients were included in the final analysis. The detailed patient selection process is illustrated in Figure 1.

### 2.2. Structural Assessment

Structural joint degeneration was evaluated using the WORMS [14], a semiquantitative scoring system that assesses cartilage integrity, bone marrow abnormalities, and other structural changes within the knee joint. Higher scores indicate greater structural degeneration. This scoring approach is widely used in research settings to characterize whole-joint structural changes and to investigate associations between imaging findings and clinical outcomes. MRI examinations were performed using a standard clinical knee imaging protocol with a 1.5-Tesla MRI system (Siemens Healthineers, Erlangen, Germany). The imaging protocol included proton density–weighted and T2-weighted sequences obtained in sagittal and coronal planes. Slice thickness ranged between 3 and 4 mm, consistent with commonly used protocols for structural knee assessment. The acquired images were subsequently evaluated using the WORMS scoring system to determine the degree of structural joint degeneration.

### 2.3. Synovial Fluid Analysis

Synovial fluid samples obtained during diagnostic or therapeutic arthrocentesis were subjected to cytological evaluation using standard laboratory procedures. The samples were examined by light microscopy to determine cellular composition [20]. Two cytological parameters were recorded: neutrophil predominance (PNL), defined as the presence of polymorphonuclear leukocyte predominance within the cellular population, and erythrocyte positivity, defined as the presence of red blood cells in the synovial fluid. These parameters were used to characterize the intra-articular inflammatory and hemorrhagic phenotype of the joint environment, consistent with commonly applied approaches in synovial fluid cytological assessment [20]. Synovial fluid samples were obtained during diagnostic or therapeutic arthrocentesis performed at the time of clinical evaluation prior to initiation of the rehabilitation program. Synovial fluid samples were processed shortly after aspiration to minimize cellular degradation. Cytological evaluation was performed using light microscopy after standard slide preparation and staining procedures routinely applied in clinical laboratories. The relative distribution of cellular components was assessed by experienced laboratory personnel. These cytological findings were recorded as categorical variables and used to characterize the intra-articular inflammatory and hemorrhagic profile of the joint environment.

### 2.4. Rehabilitation Protocol

All patients participated in a structured rehabilitation program tailored to clinical presentation and functional status. The rehabilitation approach included pain management strategies, range-of-motion exercises, muscle strengthening (particularly quadriceps-focused exercises), and progressive functional training. Rehabilitation protocols were supervised by experienced physiotherapists and adjusted according to individual progress and tolerance. The rehabilitation program was implemented in a progressive manner. The early phase focused on pain control, reduction in joint stiffness, and restoration of range of motion through gentle mobilization and stretching exercises. The intermediate phase included progressive strengthening exercises targeting the quadriceps, hamstrings, and surrounding stabilizing muscles, combined with proprioceptive training. In the later stages, functional training and activity-specific exercises were gradually introduced to improve joint stability and functional capacity. Exercise intensity and progression were individualized according to patient tolerance and clinical response.

The rehabilitation program generally consisted of supervised sessions performed 2–3 times per week for approximately 8–12 weeks, depending on clinical severity and patient progress. Each session lasted approximately 45–60 min and included a combination of pain management modalities, range-of-motion exercises, stretching, muscle strengthening, proprioceptive training, and gradual functional exercises. Progression to more advanced exercises was based on pain tolerance, restoration of joint range of motion, improvement in muscle strength, and achievement of functional milestones during follow-up. Although the overall rehabilitation framework was standardized across patients, exercise intensity, progression rate, and specific exercise selection were individualized according to patient characteristics, symptom severity, and physiotherapist assessment. Adherence to the rehabilitation program was monitored through routine outpatient follow-up visits and physiotherapist records.

### 2.5. Outcome Measures

Pain intensity was assessed using the Visual Analog Scale (VAS), a validated 0–10 scale, at three follow-up time points: 3 months, 6 months, and 1 year after initiation of rehabilitation. The primary outcome was longitudinal change in VAS scores over one year. Secondary outcomes included the association between synovial characteristics, structural degeneration (WORMS score), and pain improvement. ΔVAS was calculated as VAS at 3 months minus VAS at 1 year (VAS3m − VAS1y), such that higher positive values indicate greater pain improvement over follow-up.

### 2.6. Statistical Analysis

Continuous variables were expressed as mean ± standard deviation (SD) and median (interquartile range, IQR), as appropriate. Categorical variables were presented as frequencies and percentages. Longitudinal changes in VAS scores across the three time points were evaluated using the Friedman test for repeated measures. Post hoc pairwise comparisons were performed using the Wilcoxon signed-rank test, with Holm correction applied for multiple testing. Between-group comparisons (trauma history, PNL status, erythrocyte status) were conducted using the Mann–Whitney U test. An exploratory multivariable linear regression model was constructed to assess predictors of pain improvement, defined as ΔVAS (VAS at 3 months − VAS at 1 year). Robust standard errors (HC3) were applied. The regression model was constructed to explore potential independent predictors of longitudinal pain improvement while adjusting for relevant clinical and synovial variables. Model assumptions were evaluated prior to analysis to ensure the validity of regression estimates. To account for the potential influence of baseline pain severity on longitudinal pain improvement, an additional exploratory regression model including baseline VAS at 3 months as a covariate was also performed. A two-sided *p* value < 0.05 was considered statistically significant. Statistical analyses were performed using IBM SPSS 21.0 for Windows (Statistical Package for the Social Sciences; IBM Corporation, Armonk, NY, USA).

## 3. Results

The baseline demographic and clinical characteristics of the study population are summarized in Table 1. A total of 59 patients were included in the final analysis. The mean age of the participants was 46.02 ± 12.87 years. The cohort consisted of both male and female patients, with a slight predominance of males. The mean body weight was 74.19 ± 12.30 kg, and the mean body mass index (BMI) was within the overweight range. A history of trauma was reported in most patients (88.1%), indicating that most meniscal tears were associated with a traumatic mechanism. Regarding tear location, medial meniscal tears were more common than lateral tears. The mean structural degeneration score assessed by WORMS was 2.58 ± 0.72, reflecting mild to moderate joint structural changes. Synovial fluid cytology revealed polymorphonuclear leukocyte (PNL) predominance in 74.6% of patients, suggesting an inflammatory intra-articular environment. In addition, erythrocyte positivity was detected in 52.5% of samples, indicating the presence of intra-articular hemorrhagic components in a substantial proportion of cases.

Longitudinal changes in pain intensity measured by the VAS across the follow-up period are summarized in Table 2. VAS scores decreased significantly over time (Friedman χ^2^(2) = 110.97, *p* < 0.001), with a very large effect size (Kendall’s W = 0.94), indicating a strong and consistent reduction in pain during follow-up. Median VAS scores decreased progressively from 5 (IQR 5–6) at 3 months to 4 (IQR 3–4) at 6 months and to 1 (IQR 1–2) at the 1-year follow-up. Holm-adjusted post hoc comparisons confirmed significant reductions between all time points, indicating a consistent and clinically meaningful improvement in pain during rehabilitation.

**Figure 2:**
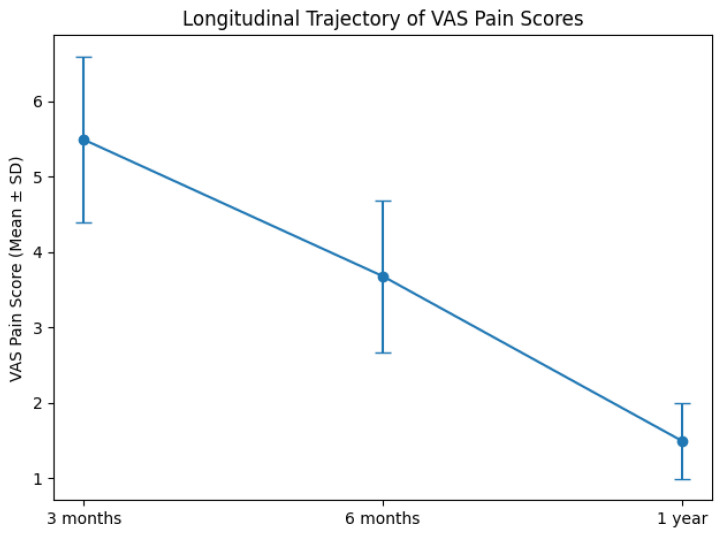
Longitudinal trajectory of VAS pain scores during follow-up. Mean VAS pain scores at 3 months, 6 months, and 1 year following rehabilitation are shown. Pain intensity decreased progressively over time (Friedman χ^2^(2) = 110.97, *p* < 0.001). Error bars represent standard deviation. Mean VAS scores (*n* = 59) ± SD were 5.49 ± 1.10 at 3 months, 3.68 ± 1.01 at 6 months, and 1.49 ± 0.50 at 1 year.

As illustrated in Figure 2, VAS pain scores demonstrated a clear and progressive decline over the follow-up period. Mean pain intensity decreased from 5.49 ± 1.10 at 3 months to 3.68 ± 1.01 at 6 months, and further to 1.49 ± 0.50 at 1 year. The downward trajectory was consistent across the cohort and statistically significant (Friedman χ^2^(2) = 110.97, *p* < 0.001), indicating sustained improvement over time. The magnitude of reduction suggests a clinically meaningful response to rehabilitation, with the most pronounced decline occurring between 3 months and 6 months, followed by continued improvement up to 1 year.

### Subgroup Analysis of Pain Outcomes Based on Clinical and Synovial Variables

Subgroup comparisons according to clinical and synovial characteristics are presented in Table 3. Patients with a traumatic etiology demonstrated significantly greater pain improvement between 3 months and 1 year (*p* = 0.041), although single time-point comparisons were not statistically significant. Neutrophil predominance (PNL positivity) was not associated with pain scores at any time point nor with longitudinal pain improvement. In contrast, erythrocyte-positive patients exhibited significantly higher pain levels at 3 months (*p* = 0.017) and greater overall pain reduction between 3 months and 1 year (*p* = 0.019). These findings suggest that intra-articular hemorrhagic features may reflect an acute inflammatory phenotype associated with higher early pain but greater subsequent recovery.

A multivariable linear regression model including age, WORMS score, PNL positivity, and erythrocyte positivity was constructed to identify independent predictors of pain improvement (ΔVAS) (Table 4). The model explained 17% of the variance in pain improvement (R^2^ = 0.17; adjusted R^2^ = 0.11) and was statistically significant (F(4, 54) = 2.80, *p* = 0.035). Erythrocyte positivity in synovial fluid was modestly associated with greater pain reduction (β = 0.75, *p* = 0.016). Age was also modestly associated with pain improvement (β = 0.025, *p* = 0.043). In contrast, WORMS score and PNL positivity were not significantly associated with pain improvement in the adjusted model. Given the modest explained variance of the model and the limited sample size, these findings should be interpreted as exploratory. An additional exploratory regression model adjusted for sex and body mass index (BMI) and for baseline VAS at 3 months yielded similar findings, with erythrocyte positivity remaining modestly associated with greater pain improvement. These results are presented in Supplementary Tables S1 and S2, respectively.

## 4. Discussion

The present study demonstrates that pain reduction following rehabilitation in patients with meniscal tears follows a consistent and clinically meaningful trajectory over one year. More importantly, our findings suggest that structural joint alterations alone may not fully explain pain behavior or recovery patterns in this cohort. Instead, synovial biological characteristics, particularly erythrocyte positivity, may represent a marker of intra-articular hemorrhagic or inflammatory activity at the time of assessment [21]. In our analysis, erythrocyte positivity in synovial fluid was modestly associated with greater pain improvement during follow-up, highlighting the potential role of intra-articular biological phenotype in recovery trajectories. However, given the relatively low explained variance and the exploratory nature of the regression model, these associations should be interpreted cautiously.

One of the most debated issues in knee pathology is the discordance between imaging findings and patient-reported symptoms [22,23]. Ota et al. [24] reported that MRI-derived structural scores, including WORMS, were not significantly associated with pain severity in knee osteoarthritis patients. Their findings support the notion that pain perception cannot be reduced to structural degeneration alone. Similarly, Elsenosy et al. [25]., in a meta-analysis of degenerative meniscal tears, demonstrated that arthroscopic surgery did not provide superior long-term pain relief compared with conservative management. These data challenge the traditional mechanistic model in which structural correction directly translates into symptomatic improvement. Consistent with this perspective, our multivariable regression analysis showed that the WORMS score, which reflects cartilage morphology and overall structural joint degeneration on MRI, was not independently associated with pain improvement. This finding suggests that structural imaging severity alone may not fully explain variability in rehabilitation outcomes within this cohort. However, given the predominantly mild-to-moderate structural degeneration observed in our sample, these results should not be interpreted as evidence that advanced structural damage lacks clinical relevance. Although much of the structural–symptom discordance literature derives from osteoarthritis populations, similar multidimensional pain mechanisms may also contribute to symptom variability in meniscal injury [26,27]. This discordance between structural findings and clinical symptoms has increasingly been interpreted within the framework of multidimensional pain models. In musculoskeletal disorders, pain perception is influenced not only by structural damage but also by inflammatory activity, neural sensitization, and psychosocial factors. As a result, patients with comparable imaging findings may experience markedly different symptom trajectories. This concept has gained particular attention in knee pathology, where structural abnormalities detected on MRI may remain clinically silent in some individuals while producing substantial pain in others. Therefore, evaluating biological indicators reflecting the intra-articular environment may provide additional insight into mechanisms underlying symptom variability.

If structural damage alone does not explain pain behavior, the intra-articular inflammatory environment becomes a central candidate [28]. Mobasheri et al. [29] introduced the concepts of “mechanoflammation” and “metaflammation,” emphasizing that mechanical stress and metabolic dysregulation can induce low-grade synovitis and perpetuate joint degeneration. In this framework, inflammation is not merely a secondary phenomenon but an active contributor to symptom generation [30]. In addition to cytokine-mediated inflammation, intra-articular hemorrhage may also contribute to the biological environment of injured joints [31]. The presence of erythrocytes within synovial fluid may indicate microvascular injury or hemarthrosis associated with meniscal trauma. Blood components introduced into the joint space can stimulate synovial irritation, promote inflammatory mediator release, and transiently amplify nociceptive signaling [32,33]. However, such processes may represent a reversible inflammatory response rather than progressive structural degeneration. This concept may partly explain the pattern observed in our cohort, where erythrocyte-positive patients demonstrated higher early pain levels but greater subsequent improvement over time [32,34].

Li et al. [35] further demonstrated that inflammatory cytokines such as IL-1β, IL-6, and TNF-α correlate more strongly with pain in earlier stages of knee degeneration, suggesting that inflammation may be particularly relevant during acute or subacute phases. Translating this to meniscal tears, erythrocyte positivity in synovial fluid may represent an acute intra-articular hemorrhagic and inflammatory phenotype [36]. In our cohort, erythrocyte-positive patients exhibited higher early pain levels but demonstrated greater pain reduction over time, and erythrocyte positivity remained a significant predictor of pain improvement in the adjusted regression model. This pattern may be consistent with a potentially non-structural pain mechanism rather than progressive structural deterioration; however, the temporal dynamics of synovial inflammation were not directly assessed in this study.

In addition to synovial characteristics, age was also identified as an independent predictor of pain improvement in the multivariable model, suggesting that patient-related factors may contribute to variability in recovery trajectories. Age-related differences in inflammatory response, tissue remodeling capacity, or rehabilitation adherence may partially explain this association [37]. Other demographic variables, including sex and body mass index (BMI), were evaluated in the adjusted analysis but were not significantly associated with pain improvement. However, given the limited variance explained by the model, these findings should be interpreted cautiously. Unlike erythrocyte positivity, neutrophil predominance (PNL) did not show a significant prognostic association. This may reflect the complexity of inflammatory cascades within the joint. As Ishijima et al. [38] highlighted, molecular and biochemical alterations may precede or operate independently of radiographic findings. Simple cytological markers may capture only part of this dynamic biological spectrum.

Another explanatory layer involves central and peripheral pain modulation. Previtali et al. [39], in a meta-analysis, reported that approximately one-fifth of patients with knee osteoarthritis exhibit features of pain sensitization, characterized by lower pain thresholds and augmented nociceptive processing. Although pain sensitization was not directly assessed in our study, the absence of a strong relationship between structural severity and pain trajectory may partially reflect neurobiological modulation mechanisms. This perspective reinforces the idea that pain recovery after meniscal injury is multidimensional, integrating structural, inflammatory, and neurophysiological components [40]. This multidimensional model better explains why some patients with similar imaging findings demonstrate divergent recovery patterns [41]. The identification of synovial erythrocyte positivity as a potential indicator of an acute inflammatory phenotype may have practical implications [42]. These findings raise the possibility that synovial characteristics could contribute to risk stratification models, although prospective validation is required before clinical implementation. Patients presenting with higher early pain but inflammatory markers suggestive of reversible processes may respond favorably to structured rehabilitation programs.

These findings may have implications for patient stratification in rehabilitation settings. Patients presenting with higher early pain levels in the presence of inflammatory synovial features may still demonstrate favorable recovery trajectories with conservative treatment [29]. Recognizing this possibility may help clinicians avoid premature surgical escalation and support the use of structured rehabilitation as an effective first-line strategy. Furthermore, synovial fluid analysis, when available in routine clinical practice, could potentially contribute to a more individualized understanding of symptom mechanisms and recovery potential [43].

This study has limitations inherent to its retrospective design. MRI rupture extent data were not available, which may limit structural characterization beyond WORMS scoring. Additionally, inflammatory biomarkers were limited to cytological findings rather than detailed cytokine profiling. Pain sensitization was not formally assessed, preventing direct evaluation of neurophysiological contributions to symptom persistence. An additional limitation of this study is that most patients had a history of trauma, indicating that the cohort predominantly represents traumatic meniscal tears. Therefore, the generalizability of these findings to degenerative meniscal tears should be interpreted with caution. Finally, the sample size, while adequate for detecting longitudinal pain changes, may limit subgroup statistical power.

## 5. Conclusions

This study demonstrates that pain reduction following rehabilitation in patients with meniscal tears occurs progressively over a one-year period and is influenced by both biological and patient-related factors. In the present cohort, synovial erythrocyte positivity and age emerged as independent predictors of greater pain improvement, whereas structural joint degeneration assessed by the WORMS score and neutrophil predominance were not significantly associated with recovery. These findings suggest that intra-articular biological characteristics may play a more important role in pain trajectories than structural imaging severity alone. The results support a multidimensional understanding of pain in meniscal injury, in which inflammatory and patient-specific factors contribute to variability in rehabilitation outcomes.

## Figures and Tables

**Table 1:** Baseline characteristics of the study population (*n* = 59).

Variable		Value
Age (years)		46.02 ± 12.87
Sex, *n* (%)	Male	34 (57.6%)
	Female	25 (42.4%)
Weight (kg)		74.19 ± 12.30
BMI (kg/m^2^)		26.4 ± 3.8
Trauma history, *n* (%)		52 (88.1%)
Tear location, *n* (%)	Medial meniscus	41 (69.5%)
	Lateral meniscus	18 (30.5%)
WORMS score		2.58 ± 0.72
PNL positive, *n* (%)		44 (74.6%)
Erythrocyte positive, *n* (%)		31 (52.5%)

Continuous variables are presented as mean ± standard deviation (SD). Categorical variables are presented as number (percentage).

**Table 2:** Longitudinal changes in VAS pain scores over one-year follow-up.

Time Point	Median (IQR)
3 months	5 (5–6)
6 months	4 (3–4)
1 year	1 (1–2)
Overall Test	Statistic
Friedman test	χ^2^(2) = 110.97, *p* < 0.001
Effect size	Kendall’s W = 0.94
Post hoc comparisons (Holm adjusted)	*p* value
3 months vs. 6 months	1.96 × 10^−9^
3 months vs. 1 year	5.09 × 10^−11^
6 months vs. 1 year	4.19 × 10^−11^

VAS values are presented as median (interquartile range). Longitudinal changes were evaluated using the Friedman test, followed by Holm-adjusted Wilcoxon signed-rank tests for pairwise comparisons.

**Table 3:** Subgroup comparisons according to clinical and synovial characteristics.

Variable	Outcome	Group 0	Group 1	*n* (0/1)	*p*-Value
Trauma history	VAS 3 months	5 (4–6)	5 (5–6)	7/52	0.065
Trauma history	VAS 1 year	2 (1–2)	1 (1–2)	7/52	0.218
Trauma history	ΔVAS (3 m → 1 y)	3 (3–4)	4 (3–4)	7/52	0.041
PNL positivity	VAS 3 months	5 (5–6)	5 (5–6)	15/44	0.861
PNL positivity	VAS 1 year	2 (1–2)	1 (1–2)	15/44	0.717
PNL positivity	ΔVAS (3 m → 1 y)	4 (3–4)	4 (3–4)	15/44	0.986
Erythrocyte positivity	VAS 3 months	5 (5–6)	6 (5–7)	28/31	0.017
Erythrocyte positivity	VAS 1 year	2 (1–2)	1 (1–2)	28/31	0.528
Erythrocyte positivity	ΔVAS (3 m → 1 y)	3 (3–4)	4 (3–4)	28/31	0.019

Data was shown as median (IQR).

**Table 4:** Multivariable linear regression analysis for predictors of pain improvement (ΔVAS).

Variable	β (Unstandardized B)	Std. Error	*p* Value
Age	0.025	0.012	0.043
WORMS score	0.170	0.215	0.433
PNL positivity	−0.070	0.340	0.841
Erythrocyte positivity	0.750	0.300	0.016

ΔVAS was defined as VAS at 3 months minus VAS at 1 year; therefore, higher positive values indicate greater pain reduction. β values represent unstandardized regression coefficients.

## Data Availability

The datasets generated and analyzed during the current study are available from the corresponding author on reasonable request. The data are not publicly available due to privacy concerns and ethical restrictions.

## Supplementary Materials

The following supporting information can be downloaded at: https://www.mdpi.com/article/10.3390/healthcare14070962/s1, Table S1: Regression model for sex and BMI, Table S2: Regression model for baseline VAS at 3 months.
