# Supplementary material for: Synovial Fluid Characteristics and Pain Recovery Trajectory Following Rehabilitation in Patients with Meniscal Tears: A Retrospective Cohort Study
Source: Healthcare (Basel). 2026 Apr 6;14(7):962. doi: 10.3390/healthcare14070962 (PMC13073586; doi:10.3390/healthcare14070962)
Supplement: Supplementary file 1 [file healthcare-14-00962-s001.zip › healthcare-4188895-supplementary.pdf]

## Supplementary Materials

To further address the potential influence of demographic characteristics on pain recovery, an additional exploratory multivariable regression model including sex and body mass index (BMI) was performed (Supplementary Table S1). The results of this extended analysis are presented in Supplementary Table S1. In this model, age remained positively associated with greater pain improvement ($\beta = 0.024$, $p = 0.049$), while erythrocyte positivity in synovial fluid continued to demonstrate a significant association with longitudinal pain reduction ($\beta = 0.731$, $p = 0.018$). In contrast, sex, BMI, WORMS score, and PNL positivity were not significantly associated with changes in VAS scores. Overall, the extended model explained a similar proportion of variance in pain improvement ($R^2 = 0.19$; adjusted $R^2 = 0.10$) compared with the primary model. These findings support the robustness of the main regression results while indicating that demographic variables did not substantially alter the observed associations.

**Table S1.** Extended multivariable linear regression model including demographic variables (sex and BMI) in addition to the primary predictors evaluated in the main analysis.

| Variable | β (Unstandardized B) | Std. Error | p value |
|---|---|---|---|
| Age | 0.024 | 0.012 | 0.049 |
| Sex (male vs female) | 0.210 | 0.310 | 0.497 |
| BMI | −0.031 | 0.028 | 0.271 |
| WORMS score | 0.158 | 0.218 | 0.472 |
| PNL positivity | −0.082 | 0.338 | 0.809 |
| Erythrocyte positivity | 0.731 | 0.302 | 0.018 |

ΔVAS was defined as the difference between VAS scores at 3 months and 1 year. β values represent unstandardized regression coefficients. Sex was coded as male = 1 and female = 0. BMI: body mass index; WORMS: Whole-Organ Magnetic Resonance Imaging Score; PNL: polymorphonuclear leukocyte predominance.

An additional exploratory regression model adjusted for baseline VAS at 3 months was performed to account for the potential influence of initial pain severity on longitudinal pain

improvement (Supplementary Table S2). In this model, baseline VAS emerged as the strongest factor associated with ΔVAS (β = 0.994, p < 0.001). After adjustment for baseline pain severity, erythrocyte positivity was no longer significantly associated with pain improvement (β = 0.087, p = 0.569). Similarly, age, WORMS score, and PNL positivity were not significantly associated with ΔVAS. These findings suggest that the greater pain reduction observed in erythrocyte-positive patients may partly reflect their higher initial pain levels rather than an independent association with recovery trajectory. Given the strong mathematical relationship between baseline VAS and ΔVAS, these findings should be interpreted cautiously

**Table S2.** Exploratory multivariable linear regression model for predictors of pain improvement (ΔVAS) adjusted for baseline VAS at 3 months.

| Variable | β | Std. Error | p value |
|---|---|---|---|
| Age | -0.001 | 0.007 | 0.877 |
| WORMS score | 0.034 | 0.103 | 0.744 |
| PNL positivity | 0.047 | 0.169 | 0.781 |
| Erythrocyte positivity | 0.087 | 0.153 | 0.569 |
| Baseline VAS at 3 months | 0.994 | 0.077 | <0.001 |

Model $R^2 = 0.831$; adjusted $R^2 = 0.815$; $F(5,53) = 43.87$, $p < 0.001$. ΔVAS was defined as VAS at 3 months minus VAS at 1 year.
